# Identification of biomarkers and potential drug targets in osteoarthritis based on bioinformatics analysis and mendelian randomization

**DOI:** 10.3389/fphar.2024.1439289

**Published:** 2024-08-29

**Authors:** Feng Cheng, Mengying Li, Haotian Hua, Ruikun Zhang, Yiwen Zhu, Yingjia Zhu, Yang Zhang, Peijian Tong

**Affiliations:** ^1^ The First School of Clinical Medicine, Zhejiang Chinese Medical University, Hangzhou, Zhejiang, China; ^2^ Department of Orthopedics, The Third Affiliated Hospital of Zhejiang Chinese Medical University, Hangzhou, Zhejiang, China; ^3^ The Third School of Clinical Medicine, Zhejiang Chinese Medical University, Hangzhou, Zhejiang, China; ^4^ Department of Gynecology, Hangzhou Women’s Hospital, Hangzhou, Zhejiang, China; ^5^ Institute of Orthopaedics and Traumatology, The First Affiliated Hospital of Zhejiang Chinese Medical University, Hangzhou, Zhejiang, China

**Keywords:** gene expression omnibus, bioinformatics analysis, mendelian randomization, osteoarthritis, biomarker, brug target

## Abstract

**Background:**

Osteoarthritis (OA) can lead to chronic joint pain, and currently there are no methods available for complete cure. Utilizing the Gene Expression Omnibus (GEO) database for bioinformatics analysis combined with Mendelian randomization (MR) has been widely employed for drug repurposing and discovery of novel therapeutic targets. Therefore, our research focus is to identify new diagnostic markers and improved drug target sites.

**Methods:**

Gene expression data from different tissues of synovial membrane, cartilage and subchondral bone were collected through GEO data to screen out differential genes. Two-sample MR Analysis was used to estimate the causal effect of expression quantitative trait loci (eQTL) on OA. Through the intersection of the two, core genes were obtained, which were further screened by bioinformatics analysis for *in vitro* and *in vivo* molecular experimental verification. Finally, drug prediction and molecular docking further verified the medicinal value of drug targets.

**Results:**

In the joint analysis utilizing the GEO database and MR approach, five genes exhibited significance across both analytical methods. These genes were subjected to bioinformatics analysis, revealing their close association with immunological functions. Further refinement identified two core genes (ARL4C and GAPDH), whose expression levels were found to decrease in OA pathology and exhibited a protective effect in the MR analysis, thus demonstrating consistent trends. Support from *in vitro* and *in vivo* molecular experiments was also obtained, while molecular docking revealed favorable interactions between the drugs and proteins, in line with existing structural data.

**Conclusion:**

This study identified potential diagnostic biomarkers and drug targets for OA through the utilization of the GEO database and MR analysis. The findings suggest that the ARL4C and GAPDH genes may serve as therapeutic targets, offering promise for personalized treatment of OA.

## 1 Introduction

Osteoarthritis (OA) is an age-related disease characterized by degeneration of articular cartilage, synovial inflammation, and subchondral bone changes leading to chronic joint pain (Grandi and Bhutani, 2020). According to the World Health Organization, 350 million people worldwide suffer from osteoarthritis. In particular, more than 50% of people over the age of 65 are affected by osteoarthritis (Chen et al., 2022a). Currently, the exact etiology of osteoarthritis is not fully understood and may be associated with factors such as aging, obesity, inflammation, trauma, metabolic abnormalities, and genetics (Englund, 2023). As a degenerative disease, there is no definitive treatment for osteoarthritis, and current treatment options mainly focus on pain relief, and the efficacy is not satisfactory (Cho et al., 2021). Therefore, the search for targets that can diagnose and treat OA remains the focus of current research. Our study hopes to further the treatment of OA by identifying new possible diagnostic markers and improving relevant drug targets.

However, the diagnosis and treatment of OA remain challenging (DeJulius et al., 2024). Previous studies have indicated that the pathogenesis of osteoarthritis primarily revolves around three aspects: synovial inflammation, cartilage degeneration, and subchondral bone sclerosis, with each scholar presenting differing viewpoints (Rai et al., 2024). While the degenerative changes in joint cartilage constitute the main pathogenic mechanism of OA, components within the joint such as the synovium and subchondral bone exhibit unique clinical manifestations. Stimulation of the joint synovium can lead to inflammation and the release of various inflammatory factors, thereby causing joint effusion (Scanzello and Goldring, 2012). Subchondral bone, located deep within the cartilage, manifests as cystic changes or osteophyte formation in different patients (Goldring and Goldring, 2010). Consequently, these three components are closely interlinked in the progression of the disease, and research focusing solely on one aspect is insufficient. Thus, our research emphasis and challenge lie in comprehensively studying the diagnosis and treatment of OA from these three perspectives.

Fortunately, with the rapid advancement of bioinformatics, we can now access gene expression data from various biological samples and experimental conditions through the Gene Expression Omnibus (GEO), a public database. This invaluable resource enables researchers to investigate gene functions, disease mechanisms, and drug treatment effects (Clough et al., 2024). We have gathered data from different tissues, including synovium, cartilage, and subchondral bone, for integration and analysis. Utilizing various bioinformatics methods, we can efficiently determine the functions and roles of differentially expressed genes, thereby providing valuable insights for our subsequent research endeavors.

Mendelian Randomization (MR) is a method used to assess causal relationships between modifiable exposures or risk factors and clinically relevant outcomes (Su et al., 2023). Because genetic variations are randomly allocated at conception, they serve as unique instruments for assessing the randomness of allocation, thus evaluating exposure-outcome causal relationships free from confounding (Ference, 2022). Studies of expression quantitative trait loci (eQTL) in disease-relevant cell/tissue types provide valuable insights into this issue by characterizing the associations between genetic variations and nearby gene expression. The Genotype-Tissue Expression (GTEx) project offers extensive eQTL data for many tissues (GTEx Consortium, Laboratory, Data Analysis & Coordinating Center (LDACC)—Analysis Working Group, Statistical Methods groups—Analysis Working Group, Enhancing GTEx (eGTEx) groups, NIH Common Fund, NIH/NCI, 2017). Genome-wide association studies (GWAS) have proven highly successful in identifying genetic variations associated with skeletal parameters (Estrada et al., 2012). Recently, research has utilized a unique osteoclast-specific eQTL dataset to identify numerous genetic regulatory effects associated with osteoporosis (Mullin et al., 2020). In the prediction of drug targets, MR analysis is also robust. A study employed MR analysis to determine the impact of lipid traits on non-alcoholic fatty liver disease (NAFLD) and explore the potential effects of lipid-lowering drug targets on NAFLD and liver functional traits (Li et al., 2023a). Conducting preliminary validation studies of differentially expressed genes obtained through the GEO database via MR analysis is an effective approach to identify candidate genes for further experimental investigation.

In this study, we identified novel therapeutic targets for OA by integrating and analyzing differential gene data in different tissues, utilizing GEO databases and Mendelian randomization methods. Bioinformatics methods were used to analyze differential gene functions, pathways and immune cell interactions. The findings were validated experimentally and the pharmacological activities of two potential OA drug targets were validated by drug prediction and molecular docking.

## 2 Materials and methods

**FIGURE 1:**
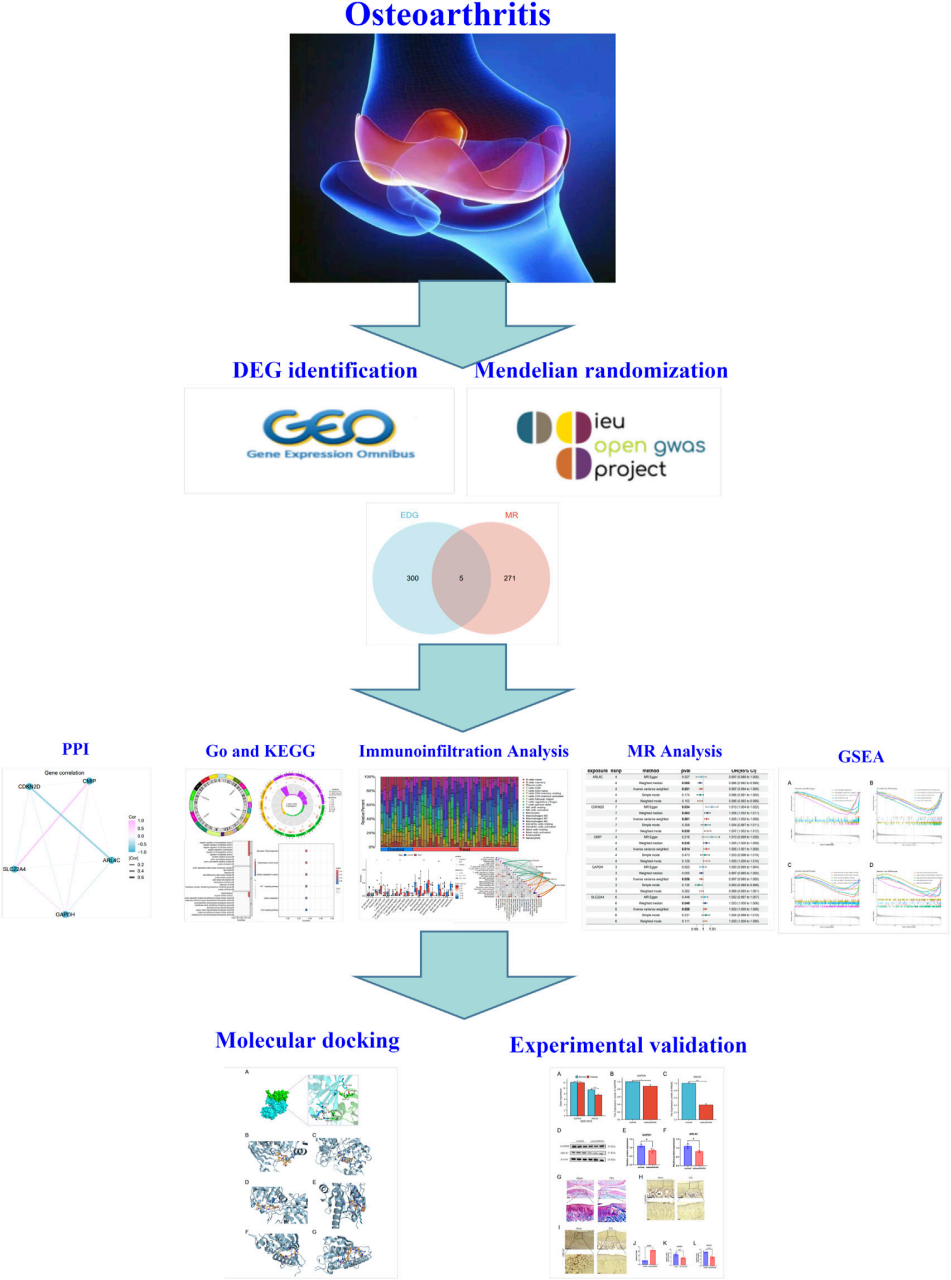
Overview of this study.

The overview design of our work was shown in Figure 1.

### 2.1 Data source

The GEO database (https://www.ncbi.nlm.nih.gov/GEO/) is a globally recognized public repository providing open access to high-throughput datasets. Through comprehensive searching and subsequent downloading, we acquired gene expression datasets related to OA disease, namely, GSE51588, GSE82107, GSE98918, and GSE10575 (Table 1). It is noteworthy that GSE51588, GSE82107, and GSE98918 served as training set datasets, while GSE10575 was employed as the validation set dataset for this study. The expression matrices of the training set datasets were integrated and batch effects were alleviated using the SVA package in R version 4.31 software.

**TABLE 1:** The data in the article comes from the GEO database.

GEO(ID)	Platform	Tissue (*Homo sapiens*)	Samples (normal/OA)	Organism	Attribute
GSE51588	GPL13497	subchondral bone	(10/40)	*Homo sapiens*	Training
GSE82107	GPL570	synovium	(7/10)	*Homo sapiens*	Training
GSE98918	GPL20844	cartilage	(12/12)	*Homo sapiens*	Training
GSE10575	GPL570	cartilage	(6/6)	*Homo sapiens*	Validation

GEO, gene expression omnibus; ID, identifier; OA, osteoarthritis.

The GWAS database (https://gwas.mrcieu.ac.uk/) is a repository providing data on global human GWAS. This database aggregates a wealth of GWAS data from various research teams, including genetic association information on diseases, physical traits, and other complex characteristics. From this database, we selected OA-related outcome data (ebi-a-GCST90038686), including 484,598 samples from Europe, of which 39,515 were cases, including data on 9,587,836 SNPS. We further chose data from the eQTLGen consortium (https://www.eqtlgen.org/) (Vosa et al., 2021) for exposure, specifically, eQTL data from 31,684 whole blood samples, for the Two-sample MR analysis.

### 2.2 Differentially expressed genes identification

Utilizing Perl and platform annotation files, probe names were converted to gene names. Background correction and normalization for each dataset were performed using the R package limma, and the sa package was employed to integrate datasets from three different anatomical sites from the same platform to mitigate batch effects (Leek et al., 2021). Differences before and after batch effect removal in samples were visualized using two-dimensional PCA clustering plots. The merged dataset was utilized as the training set for subsequent analysis.

In the R programming language, the limma package was utilized to analyze gene expression differences between normal and OA samples, based on Bayesian computation of t-values, f-values, and log odds ratios. Subsequently, differentially expressed genes (DEGs) meeting specific criteria (|logFC| ≥ 0.585 and adjusted *p*-value <0.05) between the two groups were identified. Visualization and interpretation of the obtained results were performed using the ggplot2 and pheatmap packages to generate volcano plots and heatmaps, respectively, providing intuitive representation of the findings.

### 2.3 Mendelian randomization

All data in this study were sourced from open-access databases. Utilizing a two-sample MR approach, genes were investigated for causal relationships with OA, with single nucleotide polymorphisms (SNPs) defined as instrumental variables (IVs). Core gene data were obtained from publicly available GWAS datasets. eQTL data were selected as the exposure factors, and instrumental variable SNPs were analyzed for correlation with OA disease using p1 = 5e-8, p2 = 5e-8, kb = 10000, and r2 = 0.001 to remove linkage disequilibrium SNPs and weak instrumental variables (F-test >10). Mendelian randomization analyses were conducted with OA disease. MR analysis was performed using the “TwoSampleMR” package, employing inverse variance weighting (IVW) to assess the relationship between gene levels and OA risk. The results were primarily analyzed using IVW, with significance set at *p* < 0.05, and genes showing inconsistent odds ratios across five statistical methods were excluded. Sensitivity analyses, including heterogeneity testing, horizontal pleiotropy testing, Leave One Out (LOO), and PRESSO, were conducted to ensure the robustness of the findings. Particular attention was given to horizontal pleiotropy testing, where results with *p* < 0.05 were removed.

### 2.4 Bioinformatics analysis

#### 2.4.1 GO and KEGG analysis

To further elucidate the biological mechanisms underlying the differentially expressed genes (DEGs), Gene Ontology (GO) analysis and Kyoto Encyclopedia of Genes and Genomes (KEGG) annotation were conducted using the “ClusterProfiler” R package (Yu et al., 2012).

#### 2.4.2 Immune cell analysis

To investigate the role of immune cells in OA pathology, the correlation and infiltration levels of 22 immune cell types in the OA disease group were assessed using CIBERSORT analysis (Chen et al., 2018).

#### 2.4.3 Gene network analysis

Gene correlation network maps are an important tool for showing gene interactions and functional associations in scientific research. First, five differential gene expression data were collected and pre-processed, followed by bioinformatics analysis to obtain key features. Next, network maps were constructed using known gene interaction data and visualized using the R package ggraph. Finally, the biological significance of the network was explained through functional annotation and pathway analysis, and the results were presented in the research report.

#### 2.4.4 GSEA analysis

Gene Set Enrichment Analysis (GSEA) is a bioinformatics tool used to interpret the significance of gene expression data. By considering patterns of variation across entire gene sets rather than individual genes, it evaluates the relevance of biological processes or pathways. GSEA is characterized by its high sensitivity and reliability and has become an indispensable analytical tool in biological research. In this study, two key differential genes were analyzed using the GSEA method, focusing on the biological functions and related pathways of these two genes in the low expression group of OA.

### 2.5 *In Vivo* and *in vitro* validation of differential expression gene

#### 2.5.1 Real-time quantitative polymerase chain reaction (RT-qPCR)

Human chondrocytes (C28/i2, RiSai, China) were purchased and cultured for 3-5 passages. Subsequently, the chondrocytes were seeded into 6-well plates at a density of 5 × 10^5 cells per well and divided into control and inflammation model groups. The control group was treated with high-glucose culture medium without the addition of fetal bovine serum, while the model group was supplemented with 10 μg/m of IL-1α. After 24 h, RNA was extracted from the chondrocytes using the SPARKeasy Cell RNA Rapid Extraction Kit (CisGenome, China). Genomic DNA was removed using the SPARKscript II RT Plus Kit (with gDNA Eraser) (CisGenome, China), followed by reverse transcription of the purified RNA into cDNA. Subsequently, the relative levels of target genes were determined using the SYBR Green method with an ABI 7500 fluorescent quantitative PCR instrument (ABI, United States). The 2^−ΔΔCt method was employed to compare the relative expression levels of the target genes to that of β-actin.

#### 2.5.2 Western blot analysis of protein expression

Proteins were extracted from RAPI lysed cells and quantified using the BCA assay kit (Cayman, China). Twenty micrograms of protein were subjected to constant voltage and constant current electrophoresis, followed by transfer of the target proteins onto PVDF membranes (Millipore, United States). The membranes were blocked with BSA (Sigma, United States) for 1 h, then incubated overnight at 4°C with diluted primary antibodies (rabbit anti-mouse ARL4C, GAPDH, ACTIN, at concentrations of 1:1000, 1:1000, 1:1000, respectively, Proteintech, China). After washing the membranes with PBST on a shaker for 20 min, they were incubated with secondary antibodies (goat anti-rabbit IgG, diluted 1:1000) at room temperature for 1 h, with ACTIN used as an internal control. Protein bands were visualized using ECL (Bio-Rad, Clarity Western ECL) and exposed, followed by densitometry analysis using ImageJ software.

#### 2.5.3 Rat arthritis model

Twenty male SD rats, specific pathogen-free (SPF) grade, weighing (200 ± 20) g, were obtained from Shanghai SLAC Laboratory Animal Co., Ltd., with production license number: SCKK (Shanghai) 2007-0005, and experiments were conducted at the Animal Experiment Center of Zhejiang Chinese Medical University. The experimental protocol was approved by the Medical Animal Experiment Ethics Committee (Approval No: IACUC-20230320-17).

The laboratory conditions for the rat experiments were as follows: temperature maintained at 22°C; humidity at 50%; a 12-h light-dark cycle; and *ad libitum* access to water and food. Following our established protocol, anterior cruciate ligament transection (ACLT) surgery was performed to induce OA in rats. Briefly, under anesthesia induced by intraperitoneal injection of 3% pentobarbital sodium (0.15 mL/100 g), a longitudinal skin incision was made on the medial side of the right knee. Using a surgical microscope, the ACL was transected through the medial approach after opening the knee joint via the patellar ligament. The rodents were randomly allocated into different groups, with 10 rats per group: sham surgery group (skin incision without ACL transection) and ACLT group. After 8 weeks, when the model was successfully established, the rats were euthanized, and their knee joints were harvested. The joints were fixed in 4% paraformaldehyde (PFA) for 5 days, followed by decalcification in 10% EDTA for 70 days. Subsequently, the joints were embedded in paraffin, and 5-μm consecutive sections were cut in the sagittal plane at the central medial compartment of the joint, yielding tissue slices for histological analysis (Chen et al., 2020).

#### 2.5.4 Immunohistochemical analysis

Following dewaxing and rehydration of the tissue sections, they were immersed in 1 × PBS for 3 min. Antigen retrieval was performed using 0.01 M sodium citrate buffer (pH 6.0) in a heat-induced antigen retrieval system for 4 h. Subsequently, rapid cooling was achieved by ice-cold water, followed by three washes with 1×PBS for 3 min each. The sections were then incubated in 0.3% Triton X-100 to enhance cell membrane permeability, followed by another wash with 1×PBS. After delineating the tissue boundaries, sections were treated with hydrogen peroxide blocking solution and incubated at room temperature for 10 min, followed by sample rinsing. Next, primary antibodies (ARL4C and GAPDH, diluted at 1:200, from Proteintech, China) were added and incubated overnight. On the following day, after washing the samples, secondary antibodies were applied and incubated at room temperature for 20 min. Subsequently, DAB chromogen staining was performed, and the reaction was stopped by immediate washing with water compared to the control group. Nuclear staining and counterstaining were then carried out, followed by slide sealing and image acquisition. At least three independent experiments were conducted for each sample, and image data was generated each time to ensure the reproducibility and robustness of the results. The positive cell rate analysis consisted of randomly selecting at least five high-power fields from each experiment and blind counting of positive stained cells by two independent observers to calculate the average positive cell rate. At least 1,000 cells were counted in each field of view to ensure statistical accuracy. Positive cell rates were calculated using Image Pro Plus software (number of positive cells/total cells × 100%).

### 2.6 Candidate drug prediction

Evaluating the interaction between proteins and drugs is crucial for determining whether target genes can serve as viable drug targets. In this study, we utilized the database from the United States Food and Drug Administration (FDA) to achieve this objective. This database contains approximately 1729 known compounds, including structural, physicochemical properties, and toxicological data. We aimed to identify potential candidate compounds through this database.

### 2.7 Molecular docking and prediction of drug targets

Molecular docking is a crucial method used to assess the binding affinity and interaction patterns between candidate drugs and target sites. In this study, protein structure files were initially downloaded from the PDB database, and preprocessing was conducted using the open-source software Auto dock Tools. Preprocessing steps included the addition of hydrogen ions and missing loops, removal of coordinating metal salts and water molecules, and calculation of amino acid charge distribution. Subsequently, molecular force fields and conformational optimization were applied to the proteins. Structure files of the FDA compound library were obtained from the PubChem website and preprocessed using Open Babel software, which involved adding hydrogen atoms, removing coordinating metal salts, and calculating molecular charge distribution. Finally, small molecules were subjected to molecular force fields and energy minimization to obtain three-dimensional conformations.

Autodock Tools were utilized to generate docking configuration files for the proteins. Initially, Blind Docking was employed to identify the most energetically favorable binding sites for each protein. The center coordinates, as well as the length, width, and height information of these sites, were stored in a configuration file. Two rounds of molecular docking were conducted. In the first round, all 1729 small molecules from the FDA compound library were docked to the specified binding sites in each configuration file (exhaustiveness = 10). Ten docking conformations were generated for each compound, and each conformation was scored based on binding free energy. Compounds were then ranked according to their binding affinities, and the 20 compounds with the lowest binding energies for each target protein were selected for further analysis. Additionally, the three compounds with the lowest binding energies for each target protein were subjected to the second round of molecular docking. In the second round, small molecules were docked to the binding sites with higher precision (exhaustiveness = 25), generating ten docking conformations for each compound. Interactions between each molecule and its respective target protein were analyzed and visualized. Through the analysis of molecular docking simulation results, drug candidates with high binding affinity and good interaction patterns can be identified, thus providing valuable insights for further drug design and experimental validation.

## 3 Results

### 3.1 GEO data processing

**FIGURE 2:**
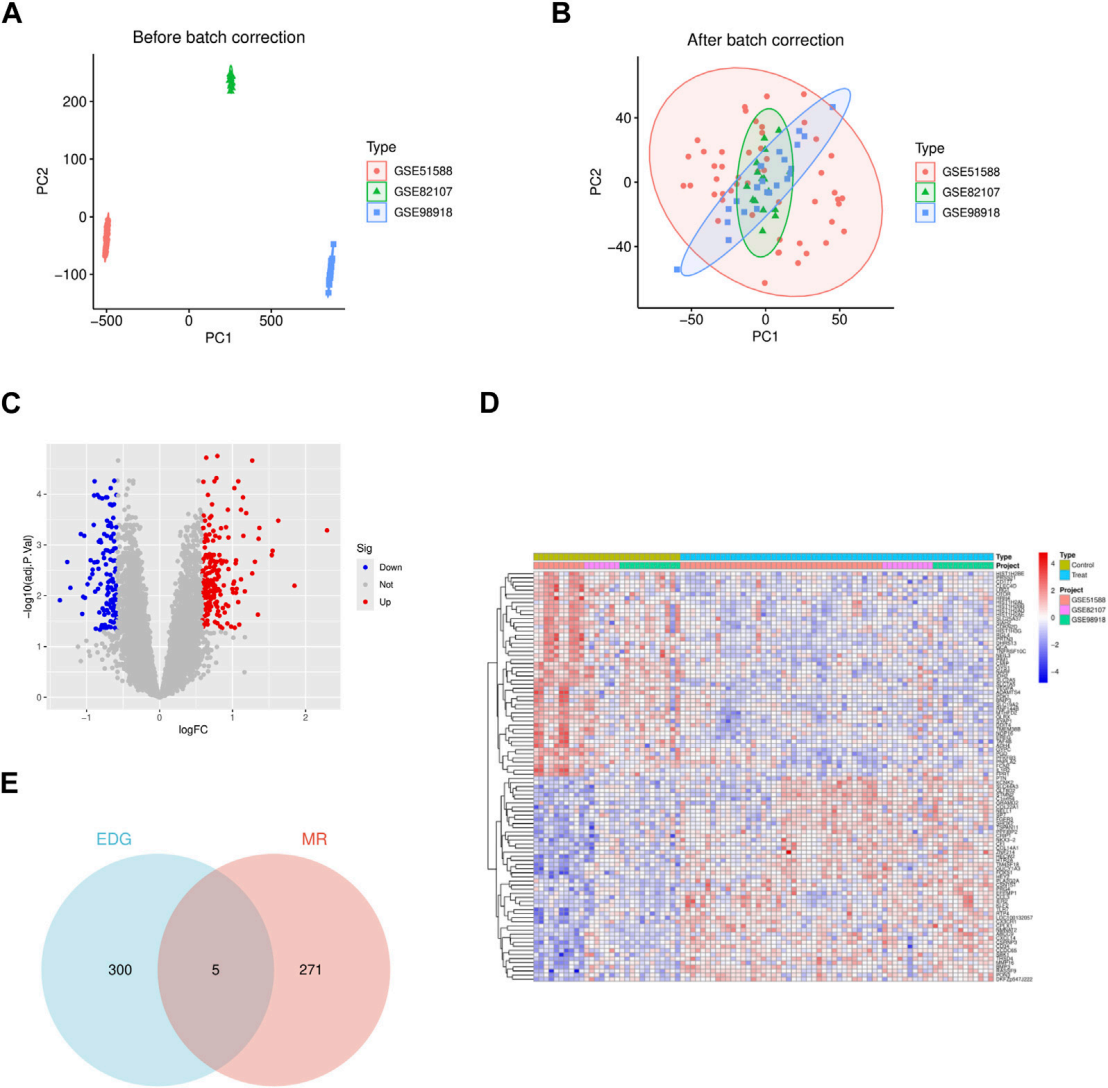
The gene differential expression analysis of GSE51588 (subchondral bone), GSE82107 (synovium), and GSE98918 (cartilage) data sets. **(A)** The two-dimensional PCA cluster plot shows the differences before the batch effect is eliminated in the sample. **(B)** The two-dimensional PCA cluster plot shows the difference after the batch effect is eliminated in the sample. **(C)** The DEG volcano map shows upregulated genes in redand downregulated genes in green. **(D)** DEG expression heat map. **(E)** The VN map shows that five of these genes are present in both sets.

We integrated three datasets, namely, GSE51588 (subchondral bone), GSE82107 (synovium), and GSE98918 (cartilage), comprising a total of 31 normal synovial samples and 62 OA samples. The gene expression levels of each sample before and after batch effect removal were analyzed, along with principal component analysis (Figures 2A, B).

### 3.2 Identification of differentially expressed genes

Using the R package limma and applying the criteria (|logFC| > 0.585 and adjusted *p*-value <0.05), we identified 305 differentially expressed genes (DEGs) associated with OA, among which 187 genes were upregulated in arthritis, and 118 genes were downregulated in OA. The detailed list of differentially expressed OA-related genes is provided in Supplementary Table S1. Heatmaps and volcano plots were employed to visualize these differences (Figures 2C, D).

### 3.3 Genome-wide MR analysis

We selected 31,684 samples from the eQTLGen consortium in whole blood as exposure and outcome data for osteoarthritis (OA) (ID: ebi-a-GCST90038686) for two-sample MR analysis. Results were primarily based on the Inverse Variance Weighting (IVW) method, with *p* < 0.05 as the threshold for statistical significance. Additionally, genes with inconsistent odds ratios across five different statistical methods and those showing horizontal pleiotropy (*p* < 0.05) were excluded. In total, 276 genes were identified to have a causal relationship with OA (Supplementary Table S2).

### 3.4 Intersection gene acquisition

We intersected the 305 DEGs obtained from GEO database analysis with the 276 genes obtained from MR analysis. The resulting intersection was visualized using a Venn diagram, revealing five genes present in both sets: SLC22A4, CDKN2D, CMIP, GAPDH, and ARL4C (Figure 2E). Sensitivity analysis conducted on these five genes yielded results consistent with the test requirements (Table 2).

**TABLE 2:** Details of sensitivity analysis of MR Results of 5 Genes and Osteoarthritis.

Exposure	Outcome	Heterogeneity tests MR-egger/IVW Cochran’s Q (pvalue)	Directional horizontal pleiotropy test	MR-PRESSO resultsGlobalTest/*p*-value
ARL4C	OA	1.573 (0.455)/1.578(0.664)	0.953	4.406/0.623
CDKN2D	OA	2.776 (0.734)/6.166(0.404)	0.124	11.720/0.354
CMIP	OA	0.918 (0.631)/2.244(0.523)	0.368	4.944/0.528
GAPDH	OA	0.075 (0.783)/1.534(0.464)	0.440	3.792/0.143
SLC22A4	OA	3.748 (0.441)/3.980(0.552)	0.655	5.183/0.723

MR, Mendelian randomization; IVW, inverse variance weighted; OA, osteoarthritis.

### 3.5 Functional enrichment analysis of intersection genes

**FIGURE 3:**
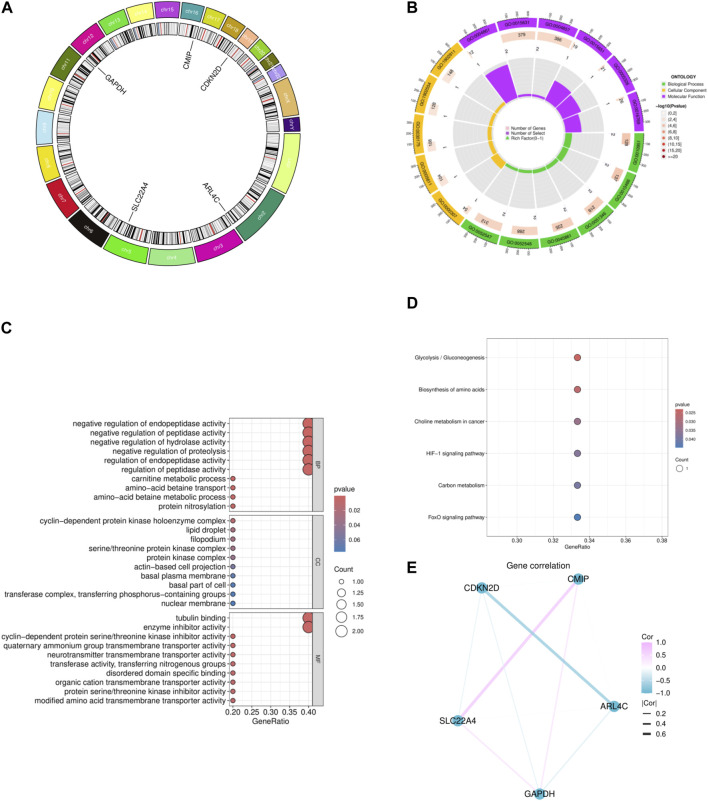
Functional enrichment analysis of five intersecting genes. **(A)** The chromosomal loop of five intersecting genes. **(B)** GO circlize map of five intersecting genes. **(C)** The bubble map of the GO of five intersecting genes. **(D)** The KEGG bubble map of five intersecting genes. **(E)** Correlation network diagram of five intersecting genes.

We conducted preliminary analysis on the five genes obtained using bioinformatics methods. The circular plot of chromosomes illustrates the locations of these five genes, namely, ARL4C, SLC22A4, GAPDH, CMIP, and CDKN2D, which are situated on chromosomes 2, 5, 12, 16, and 19, respectively (Figure 3A). From our analysis, it is evident that these genes are involved in various biological processes, including protein nitrosylation, amino acid betaine metabolism, betaine transport, carnitine metabolism, regulation of peptidase activity, regulation of endopeptidase activity, and negative regulation of protein hydrolysis. Cellular components encompass nuclear envelope, transferase complex for transferring phosphate groups, cellular basal part, basal lamina, actin-based cell protrusions, and lipid droplets. Molecular functions relate to the biochemical activity and function of gene products, including modulation of amino acid transporter activity across membranes, serine/threonine kinase inhibitor activity, and organic cation transporter activity (Figures 3B, C). Through enrichment analysis of several gene metabolic pathways and signaling pathways, we identified their crucial roles in cell biology and metabolism, involving regulation of cellular metabolism, growth, stress response, and various physiological processes related to disease occurrence and development. These include the vital role of the FoxO signaling pathway in regulating cell metabolism and growth, the central role of carbon metabolism in cellular energy production and biosynthesis, and the critical regulation of the HIF-1 signaling pathway under hypoxic conditions. Additionally, the role of choline metabolism in cancer, the importance of amino acid biosynthesis, and the regulation of glycolysis/gluconeogenesis on blood glucose levels and energy metabolism are highlighted in the figure (Figure 3D). Finally, upon examining potential interactions among the genes, we observed a connection between GAPDH and the other four genes (Figure 3E).

### 3.6 Immunocellular analysis

**FIGURE 4:**
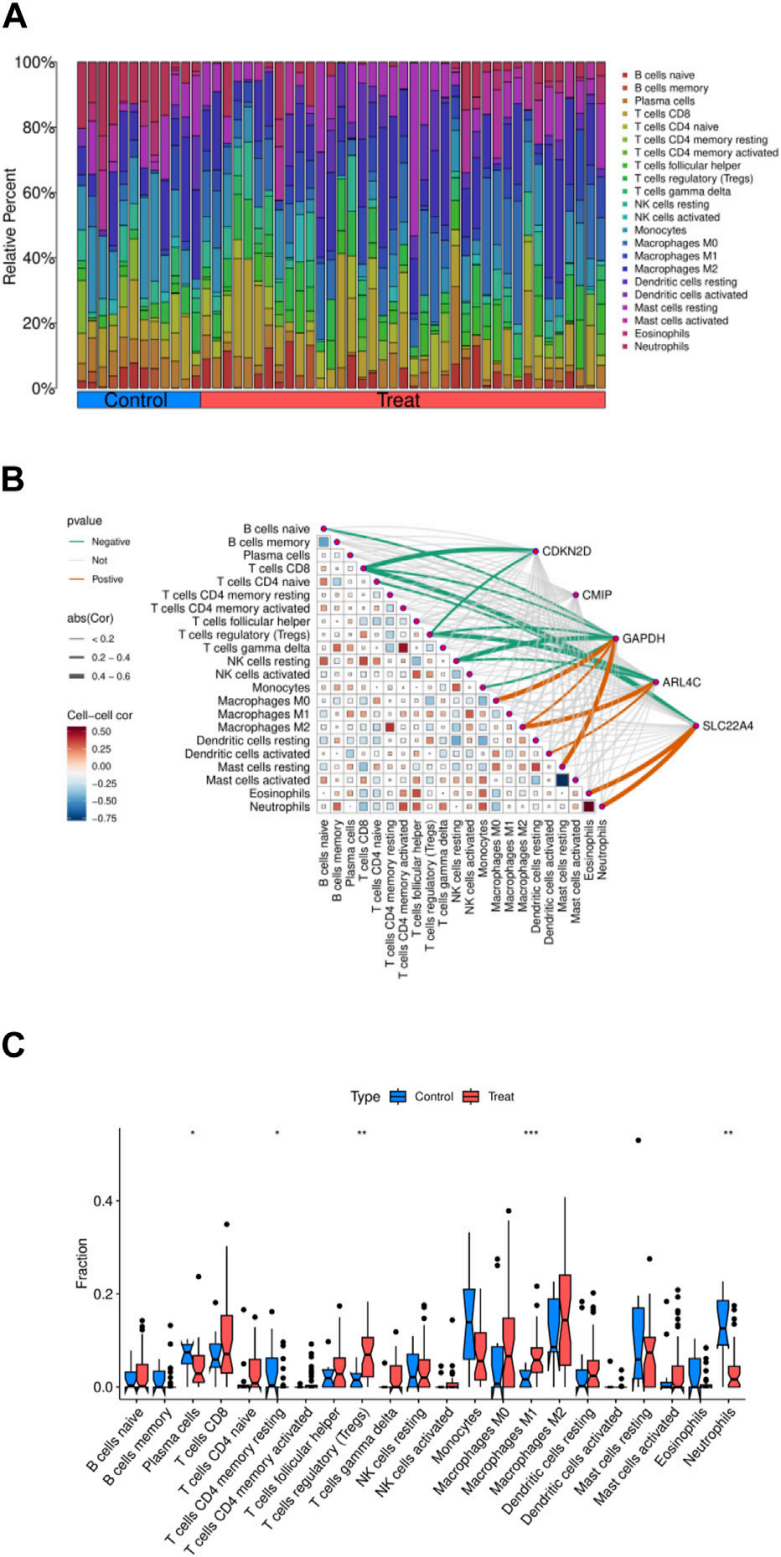
Immunoinfiltration analysis of five intersection genes. **(A)** The amount of 22 immune cells in the normal group and the experimental group. **(B)** Correlation analysis of five core genes and immune cells. **(C)** The difference of immune cell expression between normal group and experimental group.

The relationship between immune cells and OA disease is significant. We conducted an analysis on core genes and 22 immune cells, initially comparing the relative percentages of different types of immune cells under control and experimental conditions. By comparing the two sets of data, we observed alterations in the immune cell populations, indicative of significant changes in the composition of immune cell populations due to experimental interventions. For instance, memory B cells, Tregs, or specific types of T cells, and macrophages showed an increase in proportion in the experimental group, suggesting that the experimental treatment enhanced immune memory, immune suppression, or inflammatory response, thereby reinforcing cell-mediated immune response and pathogen clearance capabilities (Figure 4A). Through correlation analysis between core genes and immune cells, we observed positive correlations between ARL4C, SLC22A4, GAPDH, and Macrophages M0, Macrophages M2, Activated Dendritic cells, Eosinophils, and Neutrophils, while being predominantly negatively correlated with T cells CD8, Tregs, and resting NK cells. CMIP showed no strong correlation with any immune cells, and CDKN2D exhibited only negative correlations with immune cells, indicating relatively high associations between ARL4C, SLC22A4, GAPDH, and immune cells (Figure 4B). In our sample data, Macrophages M1, Neutrophils, and Regulatory T cells demonstrated statistical significance (Figure 4C). ARL4C, SLC22A4, and GAPDH may also regulate immune memory and immune suppression by affecting the proliferation and apoptosis of immune cells, thus modulating macrophage polarity to control inflammatory responses.

### 3.7 MR analysis of individual genes

**FIGURE 5:**
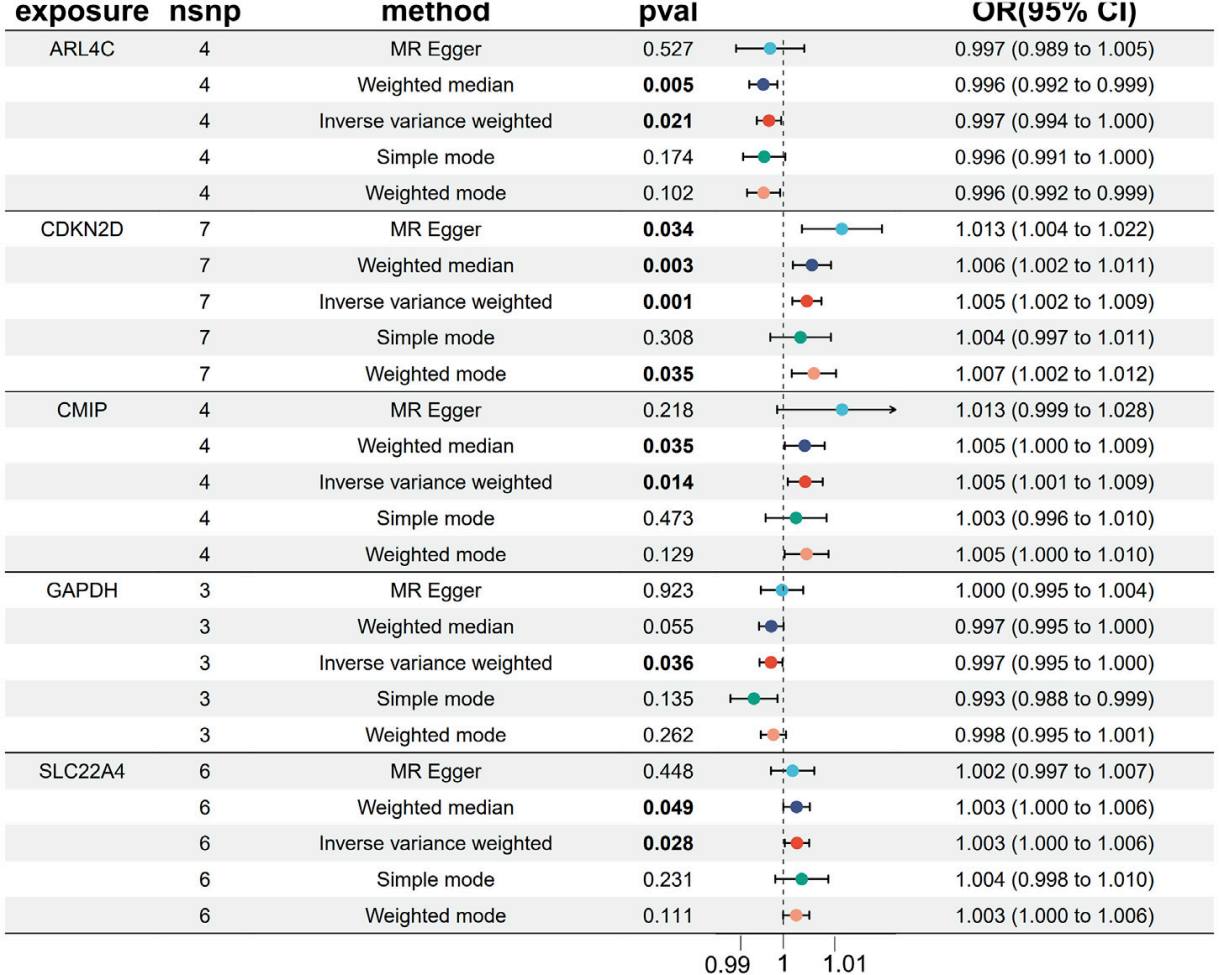
MR Forest map of five core genes and OA.

We conducted two-sample MR analyses using five genes as exposures and OA as the outcome. Our findings reveal that three genes, namely, SLC22A4, CDKN2D, and CMIP, exhibit promotive effects on OA disease, while two genes, GAPDH and ARL4C, demonstrate protective effects (Figure 5). Upon analysis of gene expression trends obtained from GEO datasets regarding OA, we observed a consistent decrease in expression levels for GAPDH and ARL4C, suggesting a protective role in OA pathology.

### 3.8 GSEA analysis of individual genes

We conducted GSEA to explore the biological functions and pathway associations of the two genes under investigation. Given the generally low expression levels of these genes in OA pathology, we focused on elucidating the biological functions and pathways associated with their low expression.

**FIGURE 6:**
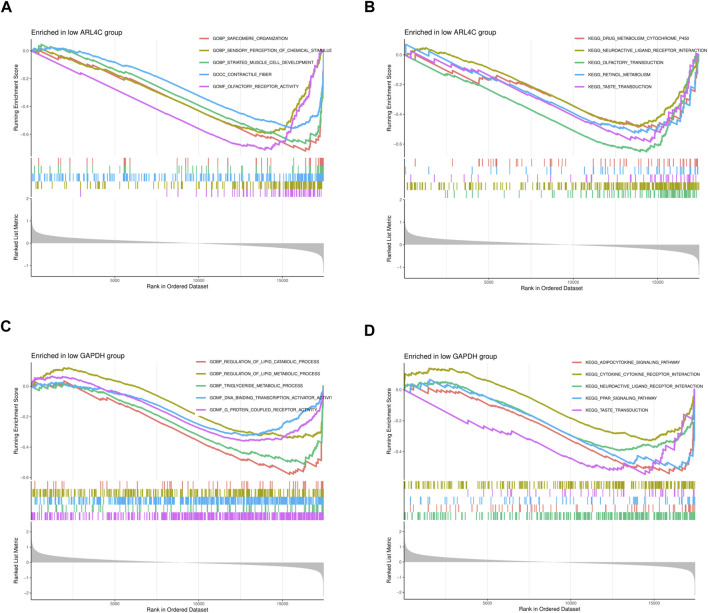
GSEA enrichment analysis of 2 DEGs. **(A)** The GO curve is enriched in the low ARL4C group. **(B)** The KEGG curve was enriched in the low ARL4C group. **(C)** The GO curve was enriched in the low GAPDH group. **(D)** The KEGG curve was enriched in the low GAPDH group.

In OA samples with decreased expression of ARL4C, functions related to muscle cell development, skeletal muscle fiber organization, chemical stimulus perception, and olfactory receptor activity were notably suppressed (Figure 6A). Additionally, ARL4C appeared to play crucial roles in processes such as drug metabolism, neurotransmitter signaling, olfaction and taste transduction, and vitamin A metabolism, indicating a potential link between reduced ARL4C expression and dysregulation in these biological processes (Figure 6B).

In OA samples with diminished expression of GAPDH, functions associated with lipid metabolism regulation, triglyceride metabolism, transcriptional activation, and GPCR signaling were inhibited. This inhibition might be related to biological phenomena such as slowed energy metabolism and alterations in cellular signaling (Figure 6C). Furthermore, our experimental findings revealed that in samples with lower GAPDH expression, pathways related to energy metabolism, immune response, neural signal transmission, and taste perception were either suppressed or altered. This observation suggests potential associations with decelerated cellular energy metabolism, changes in immune system function, abnormalities in neural regulation, and alterations in taste perception (Figure 6D).

### 3.9 Validation of gene expression in the validation set

**FIGURE 7:**
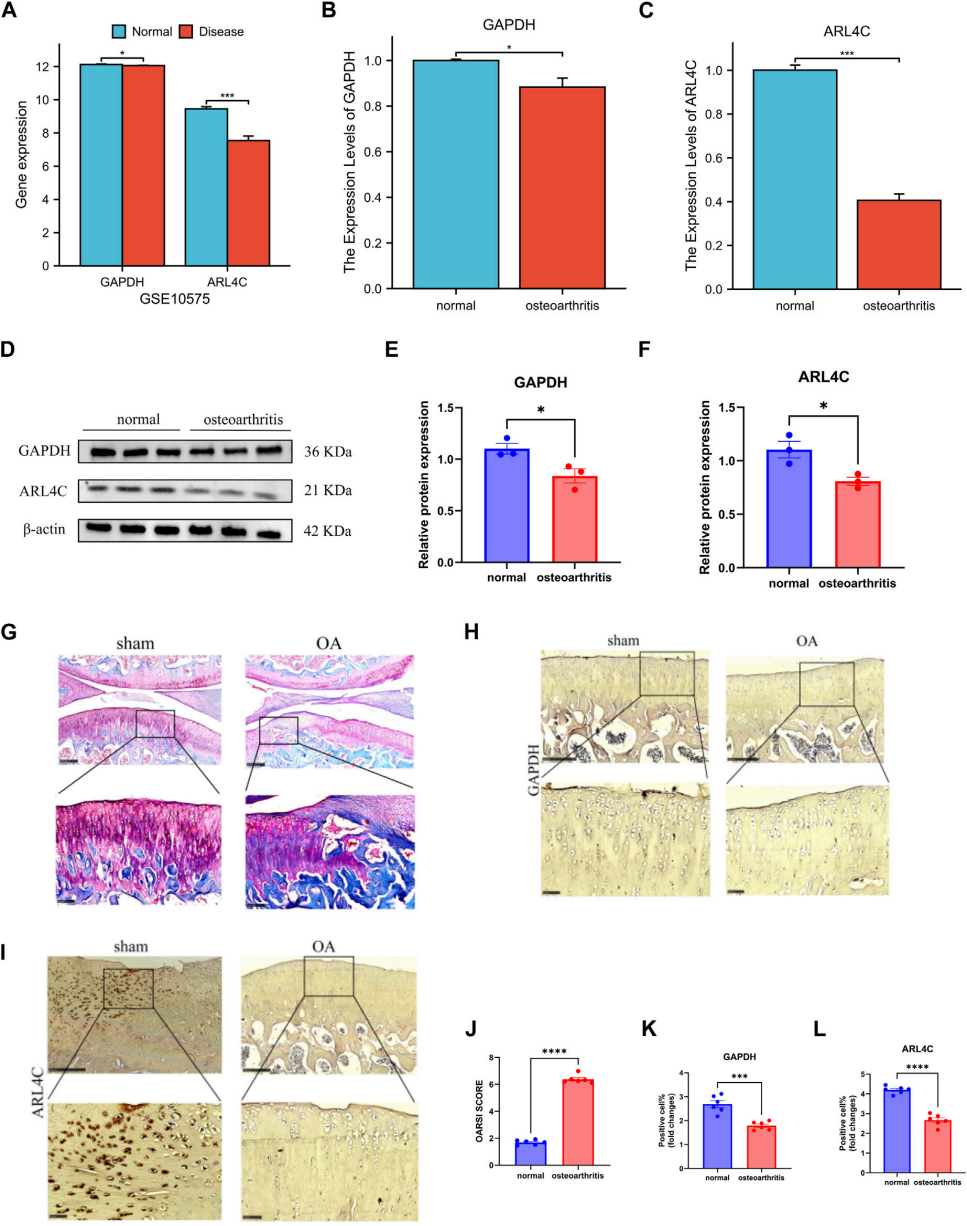
Validation set and *in vitro* and *in vivo* molecular experiments verified the results. **(A)** The expression of GAPDH and ARL4C decreased significantly in GSE10575. **(B)** The expression of GAPDH in OA samples was significantly decreased. **(C)** The expression of ARL4C in OA samples was significantly decreased. **(D)** Western blot results showed that the expression of GAPDH and ARL4C protein in OA samples was significantly decreased. **(E)** The expression of GAPDH protein in OA samples was significantly decreased. **(F)** The expression of ARL4C protein in OA samples was significantly decreased. **(G)** The stained section of the joint model group of OA rats and the sham operation group. **(H)** Immunohistochemical results of GAPDH in knee joint sham operation and OA group of rats. **(I)** Immunohistochemical results of ARL4C in knee joint sham operation and OA group of rats. **(J)** OARSI SCORE of knee OA group was significantly increased. **(K)** The positive cell rate of GAPDH in OA group was significantly reduced. **(L)** The positive cell rate of GAPDH in OA group was significantly reduced. (*) *p* < 0.05, (**) *p* < 0.01, (***) *p* < 0.001, (****) *p* < 0.0001.

In the GSE10575 dataset, we examined the expression levels of ARL4C and GAPDH genes and obtained results consistent with our expectations. The expression levels of these two genes in the cartilage of OA patients were relatively low compared to healthy individuals, indicating a protective role of these genes in the disease. Furthermore, their expression decreased progressively with disease advancement (Figure 7A).

### 3.10 Molecular experiments and *in vivo*/*in vitro* validation results

In our experimental setup, we further validated our findings. From the qPCR results, we observed varying degrees of downregulation in the expression of both genes in the chondrocyte group of normal individuals compared to the OA group, suggesting that the occurrence and progression of OA can diminish their expression levels (Figures 7B, C). Western blot analysis revealed a decrease in the expression of these two genes when translated into proteins in both normal and OA groups, further confirming our findings (Figures 7D–F). After validating our findings *in vitro*, we proceeded with *in vivo* validation. By comparing the OA rat joint model group with the sham surgery group, staining slices with safranin O-fast green showed successful construction of our model (Figures 7G, J). Immunohistochemical results of rat knee joints demonstrated a significant reduction in the expression of these two proteins, ARL4C and GAPDH, in the OA group compared to the normal group, providing further validation of our findings *in vivo* (Figures 7H, I, K, L).

### 3.11 Prediction of candidate drugs and molecular docking analysis

**FIGURE 8:**
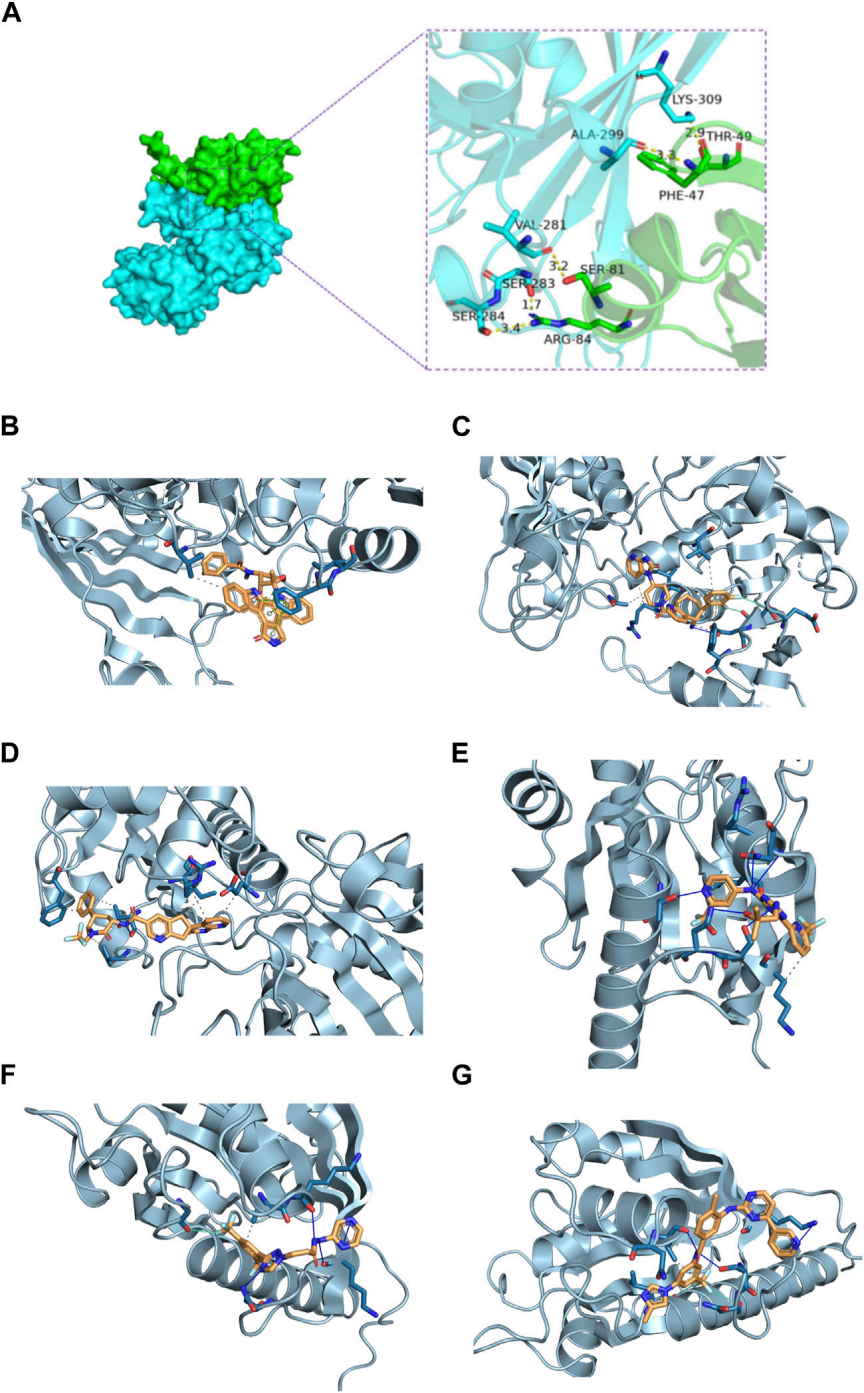
Molecular docking of GAPDH and ARL4C proteins and prediction of drug targets. **(A)** The GAPDH and ARL4C proteins form complexes through hydrogen bonding of surrounding residues. **(B)** Molecular docking diagram of GAPDH and Rimegepant. **(C)** Molecular docking diagram of GAPDH and Midostaurin. **(D)** Molecular docking diagram of GAPDH and Ubrogepant. **(E)** Molecular docking diagram of ARL4C and Enasidenib. **(F)** Molecular docking diagram of ARL4C and KPT330. **(G)** Molecular docking diagram of ARL4C and Nilotinib.

In this section, we explored the potential interaction between GAPDH and ARL4C proteins. The docking results revealed a binding energy of −275.41 kcal/mol for the GAPDH-ARL4C complex. Residues surrounding the protein-protein interaction interface were found to form hydrogen bonds, contributing to the stabilization of the protein-protein complex. Specifically, ARG-84, SER-81, PHE-47, and THR-49 of ARL4C were observed to form hydrogen bonds with SER-284, SER-283, VAL-281, ALA-299, and LYS-309 of GAPDH, with bond lengths ranging from 1.7Å to 3.4Å, indicating potential active residues (Figure 8A).

Subsequent screening of the FDA library identified the top three small molecules with the highest scores for binding to GAPDH: Ubrogepant, Midostaurin, and Rimegepant, with binding energies of −12 kcal/mol, −11.9 kcal/mol, and −11 kcal/mol, respectively (Table 3; Figures 8B–D). Additionally, the top three small molecules capable of binding to ARL4C were identified as Enasidenib, Nilotinib, and KPT330, with binding energies of −8.1 kcal/mol, −8 kcal/mol, and −7.4 kcal/mol, respectively (Table 3; Figures 8E–G). These findings suggest potential candidates for further investigation as therapeutic agents against osteoarthritis.

**TABLE 3:** Docking results of two proteins with small molecules.

Target	Structure ID	Drug	PubChem	Binding energy (kcal/mol)
ARL4C	ARL4C (AlphaFold)	Enasidenib	89683805	−8.1
		Nilotinib	644241	−8
		KPT330	71481097	−7.4
GAPDH	1U8F (PDB)	Ubrogepant	68748835	−12
		Midostaurin	9829523	−11.9
		Rimegepant	51049968	−11

The lower the Binding Energy, the better the binding efect and the higher the afnity.

## 4 Discussion

Regarding research on OA, early scholars mainly focused on cartilage wear and degeneration. However, with deeper investigation, OA is now regarded as a systemic joint disease. It involves interactions among articular cartilage, synovium, subchondral bone, ligaments, muscles, and so forth (Loeser et al., 2012). The interplay among synovium, cartilage, and subchondral bone accelerates joint cartilage damage and loss, as well as the occurrence of structural abnormalities and low-grade chronic inflammation in the subchondral bone (Butterfield et al., 2021). Evidence suggests a unique regulatory relationship between cartilage and subchondral bone. Experiments on small molecule diffusion have revealed the presence of direct molecular signaling between cartilage and subchondral bone, which may contribute to OA progression (Hu et al., 2021). Relevant studies indicate that osteoarthritis is best conceptualized as a disease of the entire “joint organ,” and the infrapatellar fat pad (IFP), a fat tissue near the synovium, interacts with the synovium, articular cartilage, and subchondral bone, accelerating OA progression (Zeng et al., 2020). A comprehensive search for core genes influencing OA diagnosis and treatment may be a crucial step in OA management (Tong et al., 2022).

In our study, we collected gene expression data from different sources, including synovium, cartilage, and subchondral bone, from the GEO database. Through combined MR analysis, we identified two genes, GAPDH and ARL4C, with consistent trends in expression levels and MR. Their expression in OA is reduced, exerting a protective effect against the disease. We not only validated these two genes in the validation set but also confirmed our findings through *in vitro* and *in vivo* experiments. We believe that these two genes may play important roles in the occurrence, development, or treatment of OA.

Glyceraldehyde-3-phosphate dehydrogenase (GAPDH) is a critically important enzyme with multifaceted functions in both biology and medicine (Mondragón et al., 2019). Its primary role lies within the glycolytic pathway, where it catalyzes the conversion of glyceraldehyde-3-phosphate (G3P) to 1,3-bisphosphoglycerate (1,3-BPG), a redox reaction essential for cellular energy production, concomitantly reducing NAD + to NADH (Liao et al., 2019). Studies indicate that GAPDH harbors an active site cysteine residue susceptible to oxidation under hydrogen peroxide, resulting in rapid enzyme inactivation, with its redox-switching activity serving to preserve reductive capacity and promote the survival of stressed tumor cells (Talwar et al., 2023). Moreover, GAPDH plays a regulatory role in gene transcription and protein translation within cells. It binds to RNA, influencing gene expression levels (Tossounian et al., 2020), and interacts with cytoskeletal proteins to maintain cellular morphology and motility, thus preserving cellular architecture (Santos et al., 2023). Recent research highlights GAPDH’s involvement in inhibiting homologous recombination to stabilize cellular morphology, achieved through protein interactions and deacetylation (Shi et al., 2023). Additionally, GAPDH is implicated in regulating apoptosis and oxidative stress; S-glutathionylation of GAPDH transmits signals to the nuclear GAPDH transglutaminase, initiating cell apoptosis (Mustafa Rizvi et al., 2021).

While the functionality of GAPDH is robust, unfortunately, there is a dearth of research on it in OA. Most scholars have not recognized the expression differences of GAPDH in OA, some even utilize GAPDH as an internal reference in molecular experiments. However, recent studies have found that the expression of GAPDH may vary under certain pathological conditions, posing challenges to its widespread use as an internal reference. The reliability assessment of GAPDH requires consideration of its expression changes in disease states. Since GAPDH expression in OA may not be constant, researchers need to exercise caution when using GAPDH as an internal reference and consider other potential reference genes or adopt more precise quantitative methods to ensure the accuracy of research results (Long et al., 2020). In recent years, mounting evidence suggests a crucial association between GAPDH and immune cells. GAPDH inhibits the increase in pH value of neutrophils, blocking this increase prevents cell death and the formation of neutrophil extracellular traps (NETs) (Li et al., 2023b). Recently, a serum acylation system of GAPDH has been reported, which promotes the glycolytic metabolism and anti-tumor immune activity of CD8 T cells (Wang et al., 2024). GAPDH reverses the activation of Th2 cells induced by M2 through its glycolytic activity, which plays an important immunomodulatory role in preventing allergic asthma (Chen et al., 2022b). Of course, GAPDH also interacts with other proteins, collectively influencing the polarization changes of macrophages. GAPDH oxidation plays a procedural role in shaping the metabolism and inflammatory characteristics during macrophage activation (Yoo et al., 2022). It is well known that OA is a low-grade inflammation and progressive joint disease, and its progression is closely related to the imbalance of M1/M2 synovial macrophages (Zhang et al., 2022). These studies provide us with some clues, but further experimental research is needed to elucidate how GAPDH affects the molecular mechanisms of OA.

The protein encoded by the ARL4C gene is a small GTPase, which is one of the members of the ARL (ADP-ribosylation factor-like) family (Zhao et al., 2023). The ARL4C protein participates in various biological processes within cells, including intracellular membrane trafficking, cell polarity, and cell signaling (Han et al., 2020). Aberrant expression or mutation of ARL4C may impact these biological processes, thereby being associated with the occurrence and development of diseases, such as endometrial cancer (Zhang et al., 2020). There are some associations and interactions between ARL4C and macrophages. Macrophages are an important cell type in the immune system, primarily involved in clearing foreign substances, bacteria, and dead cells to maintain tissue cleanliness and immune balance (Ji et al., 2010). Some studies suggest that ARL4C may regulate macrophage polarity, migration, and phagocytic functions (Lazarov et al., 2023). Additionally, ARL4C has been studied in other cell types as well. For instance, ARL4C expression dependent on the RAF1-MEK/ERK pathway promotes ameloblastoma cell proliferation and osteoclast formation (Fujii et al., 2022). ARL4C also plays a role in drug targeting; miR-26 promotes foam cell formation by reducing ABCA1 and ARL4C expression, supporting the development of drugs targeting atherosclerosis (Chen et al., 2024). In lung cancer research, the ubiquitination regulation mechanism of Arl4c is studied, and potential chemotherapeutic drugs targeting Arl4c are explored (Sun et al., 2020). Although most studies on ARL4C currently come from tumors, its biological functions and role in macrophage polarization provide us with some directions for studying OA. In our research, ARL4C is identified as a protective gene in OA, with decreased expression in OA diseases. It may serve as a good diagnostic marker and drug target, and increasing the level of ARL4C in OA patients may have a certain therapeutic effect.

In the field of OA, a significant unmet need remains the development of reliable phenotyping and stratification capabilities for patients, enabling more effective targeted therapies for inclusion in clinical trials (Winthrop et al., 2024). Therefore, the identification of essential genes is imperative, as it provides insights into core structures and functions, expediting the discovery of drug targets and other functionalities (Aromolaran et al., 2021). Molecular docking aids researchers in understanding the interactions between drugs and target molecules, facilitating high-throughput screening from databases (Li et al., 2022).

In our study, we initially predicted the potential interaction between two genes. Surprisingly, we found that GAPDH and ARL4C can form a protein-protein complex through surrounding residues, potentially stabilized by hydrogen bonds. Whether their complex influences the development of OA disease by modulating macrophage polarization remains speculative. Subsequently, we conducted two rounds of screening on the FDA library via virtual docking, predicting potential small-molecule compounds that could target GAPDH and ARL4C. Drug prediction and molecular docking were utilized to validate the pharmacological value of these targets. These findings offer promising clues for more effective OA therapies, potentially reducing drug development costs and advancing personalized medicine approaches. This research makes a valuable contribution to the field, emphasizing the importance of these identified targets in OA treatment.

While our study contributes valuable insights, it is essential to acknowledge its limitations. Firstly, despite the pivotal role of the GEO as a public repository for gene expression data, it presents various challenges. These include disparities in data quality and consistency, uneven distribution and coverage of data, lack of standardization in data format and annotation, inadequate metadata, delayed data updates and maintenance, and ethical concerns regarding data privacy. These issues may undermine the reliability of our conclusions.

Secondly, while MR serves as a causal inference method based on genetic instrumental variables, facilitating the determination of causal relationships between variables (Bowden and Holmes, 2019). However, this approach has limitations. Mendelian randomization relies on the analysis of existing datasets and is therefore unsuitable for newly emerged or unobservable variables. To accurately establish causal relationships between variables, a combination of methods is necessary, corroborating evidence from various sources (Liu et al., 2023). Moreover, the majority of datasets predominantly represent European populations, lacking diversity in race and gender in GWAS. Disparities in population backgrounds, influenced by genetic backgrounds and linkage disequilibrium patterns, may introduce potential biases in MR effect estimates. Additionally, reliance on blood-based eQTL for MR testing poses challenges in identifying the most effective tissues for therapeutic interventions. Different tissues may exhibit distinct genetic regulatory mechanisms, and focusing solely on blood eQTL may not provide a comprehensive understanding of diseases and potential treatment modalities.

Furthermore, our *in vitro* and *in vivo* experiments were kept straightforward, focusing solely on validation at the expression level. We utilized specimens of human chondrocyte inflammatory models and rat arthritis models, without collecting clinical samples for validation. Gene function was not verified through downregulation or overexpression to observe phenotypic changes in OA, nor were experiments conducted on transgenic mice. Additionally, the accuracy of molecular docking analysis heavily relies on the quality of protein structures and ligands. While this method identifies potential drug targets, it does not guarantee their effectiveness in clinical settings. Subsequent experimental validation and clinical trials are necessary to confirm the therapeutic potential of the identified targets.

## 5 Conclusion

In this study, the potential diagnostic biomarkers and drug targets for OA were identified using a combination of the GEO database and MR analysis. It was observed that ARL4C and GAPDH genes exhibited significance in both cohorts and were supported by *in vitro* and *in vivo* molecular experiments. These genes are associated with macrophage polarization function and may serve as effective therapeutic targets for OA. Additionally, drug prediction and molecular docking were employed to validate the pharmacological value of these targets. These findings provide promising leads for more effective diagnosis and treatment of OA, potentially reducing drug development costs and advancing personalized medicine approaches.

## Data Availability

The original contributions presented in the study are included in the article/Supplementary Material, further inquiries can be directed to the corresponding author.
