# Supplementary material for: Identification of biomarkers and potential drug targets in osteoarthritis based on bioinformatics analysis and mendelian randomization
Source: Front Pharmacol. 2024 Aug 29;15:1439289. doi: 10.3389/fphar.2024.1439289 (PMC11390638; doi:10.3389/fphar.2024.1439289)

**
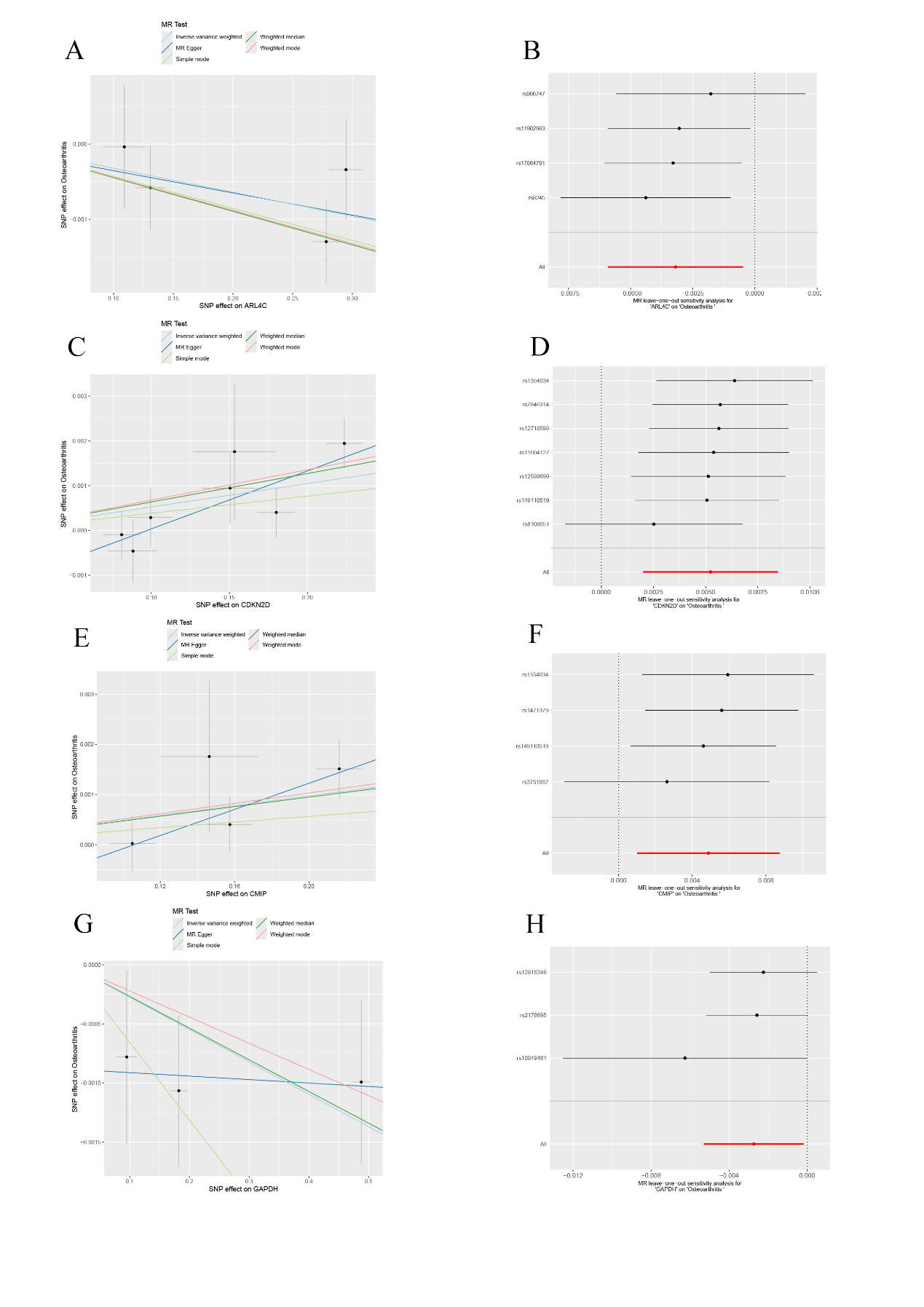
 Figure S1.** MR Plots for Relationship of Five core genes with osteoarthritis.


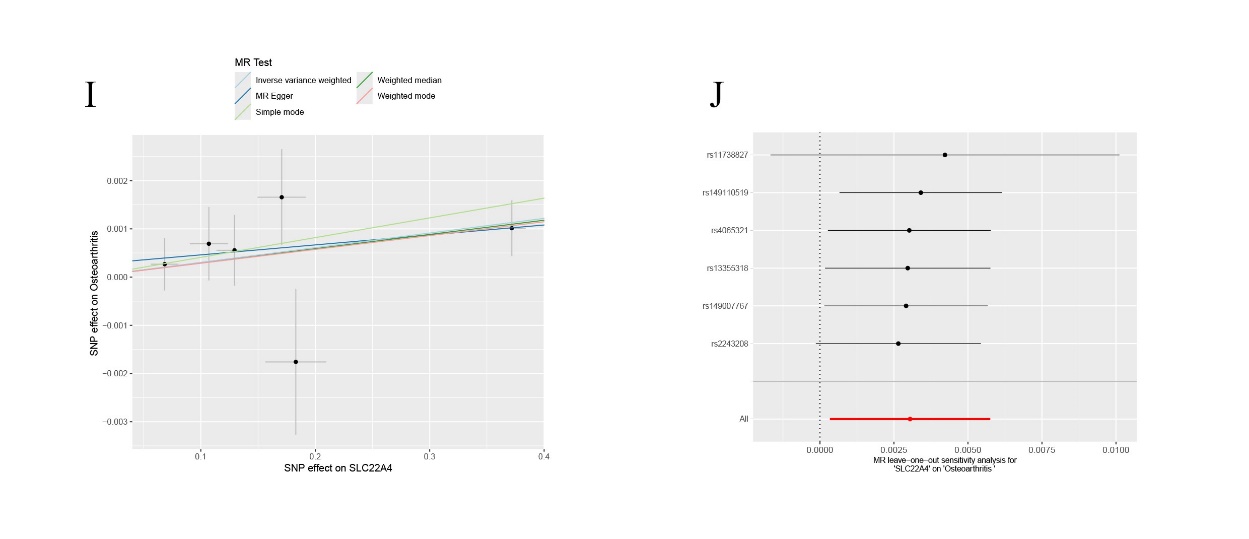


**Figure S2.** the full uncropped Gels and Blots image(s) of tow proteins


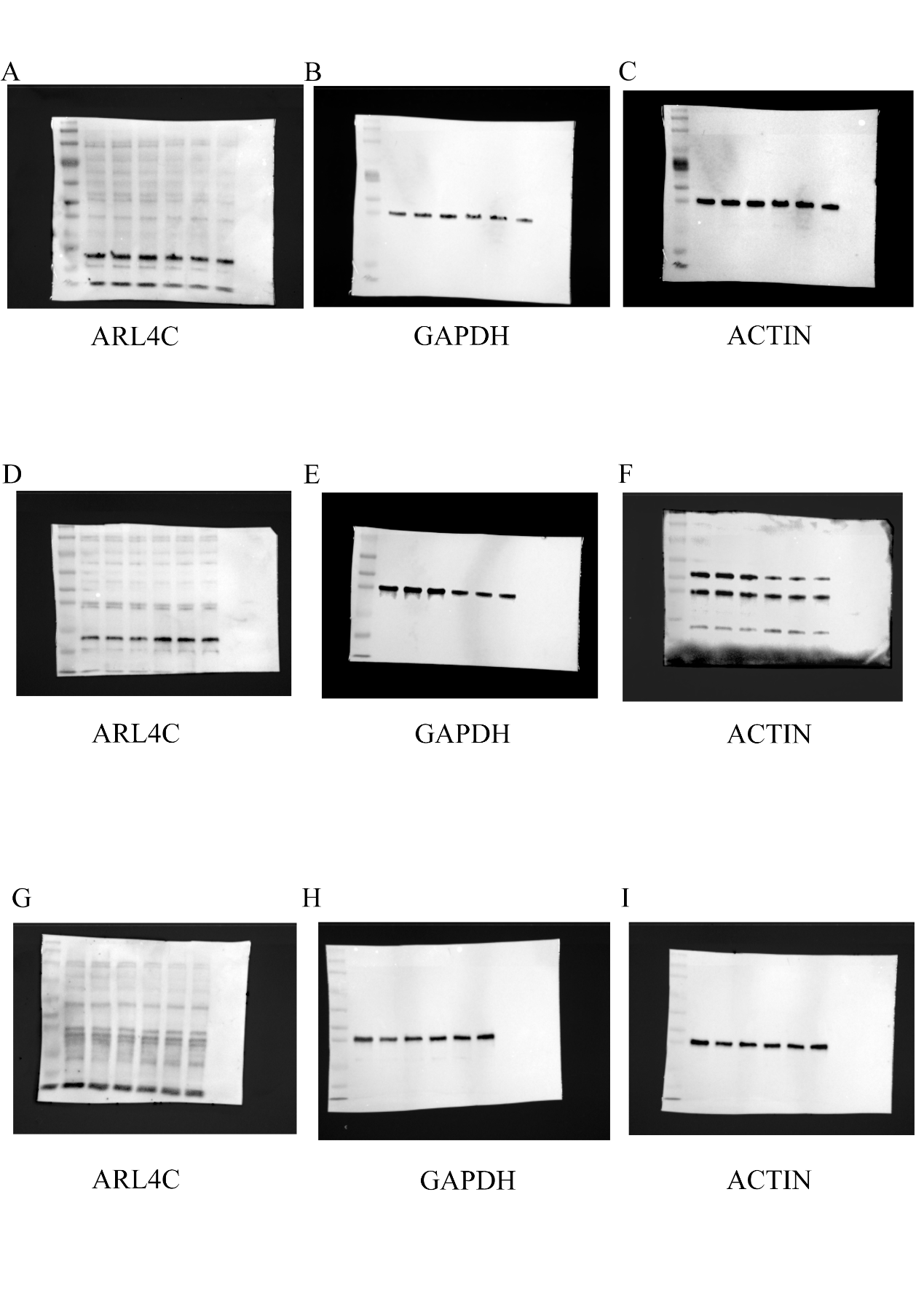

Supplement: Supplementary file 3 [file DataSheet1.docx]
